# Efficacy of Prostacyclin Anticoagulation in Critically Ill Patients Requiring Extracorporeal Support: A Systematic Review and Meta-Analysis

**DOI:** 10.7759/cureus.39967

**Published:** 2023-06-05

**Authors:** Nedaa Aldairi, Alyaa S Al Ali, Muneera Alabdulqader, Majed Al Jeraisy, John Cyrus, Oliver Karam

**Affiliations:** 1 Pediatric Critical Care, Dr. Sulaiman Al Habib Medical Group, Riyadh, SAU; 2 Pediatric Critical Care, Sheikh Khalifa Medical City, Abu Dhabi, ARE; 3 Pediatrics, King Faisal University, Al Ahsa, SAU; 4 Clinical Pharmacy, King Abdullah International Medical Research Canter, King Saud Ben Abdulaziz University for Health Sciences, Riyadh, SAU; 5 Health Sciences Library, VCU Libraries, Virginia Commonwealth University, Richmond, USA; 6 Pediatric Critical Care, Department of Pediatrics, Yale School of Medicine, New Haven, USA

**Keywords:** meta-analysis, continuous renal replacement, mortality, thrombosis, hemorrhage, anticoagulant, critical illness, prostaglandin i

## Abstract

Extracorporeal support modalities are highly prothrombotic. Anticoagulation is frequently used for patients receiving Continuous Renal Replacement Therapy (CRRT), Molecular Adsorbent Recirculating System (MARS), and Extracorporeal Membrane Oxygenation (ECMO). The objective of this systematic review and meta-analysis is to determine if prostacyclin-based anticoagulation strategies are effective compared to other anticoagulation strategies, in critically ill children and adults who needs extracorporeal support, such as continuous renal replacement therapy.

We conducted a systematic review and meta-analysis using multiple electronic databases and included studies from inception to June 1, 2022. Circuit lifespan, proportion of bleeding, thrombotic, and hypotensive events, and mortality were evaluated. Out of 2,078 studies that were screened, 17 studies (1,333 patients) were included. The mean circuit lifespan was 29.7 hours in the patients in the prostacyclin-based anticoagulation series and 27.3 hours in the patients in the heparin- or citrate-based anticoagulation series, with a mean difference of 2.5 hours (95%CI -12.0;16.9, p=0.74, I^2^=0.99, n=4,003 circuits). Bleeding occurred in 9.5% of the patients in the prostacyclin-based anticoagulation series and in 17.1% of the patients in the control series, which was a statistically significant decrease (LogOR -1.14 (95%CI -1.91;-0.37), p<0.001, I^2^=0.19, n=470). Thrombotic events occurred in 3.6% of the patients in the prostacyclin-based anticoagulation series and in 2.2% of the patients in the control series, which was not statistically different (LogOR 0.97 (95%CI -1.09;3.04), p=0.35, I^2^=0.0, n=115). Hypotensive events occurred in 13.4% of the patients in the prostacyclin-based anticoagulation series and in 11.0% of the patients in the control series, which was not statistically different (LogOR -0.56 (95%CI -1.87;0.74), p=0.40, I^2^=0.35, n=299). The mortality rate was 26.3% in the prostacyclin-based anticoagulation series, and 32.7% in the control series, which was not statistically different (LogOR -0.40 (95%CI -0.87;0.08), p=0.10, I^2^=0.00, n=390). The overall risk of bias was low to moderate.

In this systematic review and meta-analysis of 17 studies, prostacyclin-based anticoagulation was associated with fewer bleeding events, but with similar circuit lifespans, thrombotic events, hypotensive events, and mortality rates. The potential benefits of prostacyclin-based anticoagulation should be explored in large randomized controlled trials.

## Introduction and background

Thrombotic complications are a frequent concern in patients receiving Continuous Renal Replacement Therapy (CRRT) [1], Molecular Adsorbent Recirculating System (MARS) [2], and Extracorporeal Membrane Oxygenation (ECMO) [3]. CRRT is a life support modality used for acute kidney injury, while ECMO is used for severe respiratory and cardiac failure. Both therapies involve the use of extracorporeal circuits in which blood comes in contact with artificial surfaces, which increases the risk of thrombosis. To prevent these complications, anticoagulation is typically administered [1,4], which should ideally minimize clot formation while also minimizing the risk of bleeding [5]. 

Most CRRT anticoagulation strategies involve systemic unfractionated heparin or regional citrate infusions, while most ECMO anticoagulation strategies involve unfractionated heparin or bivalirudin [1,5]. These strategies target the coagulation cascade but fail to address another part of hemostasis, the platelet function.

Prostacyclin or prostaglandin I2 (PgI2), a prostaglandin member of the eicosanoid family of polyunsaturated fatty acids, inhibits platelet activation and is an effective vasodilator. While prostacyclin is widely used to treat pulmonary hypertension, its antiplatelet effect is seldom sought [6]. Since this molecule has a different mechanism than heparin, citrate, or bivalirudin, its benefit-to-risk ratio might be valuable in critically ill patients requiring extracorporeal support. However, there is limited evidence regarding its safety, efficacy, and impact on patient outcomes [7,8] in this population.

The objective of this systematic review and meta-analysis is to compare the efficacy of prostacyclin-based anticoagulation strategies (either PgI2 alone or in association with another anticoagulant) with other anticoagulation strategies in critically ill patients requiring extracorporeal support. 

## Review

Methods

Design

This is a systematic review of observational studies or randomized trials to determine the duration of the extracorporeal circuit, the prevalence of bleeding, thrombosis, thrombocytopenia, hypotension, and mortality, according to the anticoagulation strategy (prostacyclin as compared to other anticoagulation strategies or no intervention), in critically ill children and adults who needs extracorporeal support. The review followed recommendations contained within the PRISMA statement [9]. This study was registered with PROSPERO (Record ID: 332326).

Types of Studies

We included all studies, including abstracts submitted to conferences, case series, retrospective or prospective controlled studies, and randomized controlled trials that 1) enrolled critically ill children and/or adults who require extracorporeal support, such as continuous renal replacement therapy (CRRT), molecular adsorbent recirculating system (MARS), and extracorporeal membrane oxygenation (ECMO); 2) that compared the use of prostacyclin with other anticoagulation strategies; and 3) that report at least one outcome of interest. We included “grey literature”, which includes abstracts of unpublished studies and studies published in non-peer-reviewed journals. We excluded studies where prostacyclin was used as a pulmonary vasodilator, studies without a comparator (i.e. prostacyclin only), studies without clinical outcomes (e.g. laboratory values only), studies outside of a critical care setting (bypass, chronic dialysis, etc.), individual case reports, case series of 10 patients or less, reviews or editorials not providing original results, and animal studies. 

Types of Participants

We included studies that enrolled subjects on extracorporeal support (CRRT, MARS, and/or ECMO). Patients were further categorized as children or adults. 

Types of Interventions

Based on the use of prostacyclin, anticoagulation strategies were categorized as intervention (either PgI2 alone or in association with another anticoagulant) or as control, when the anticoagulation strategy did not include PgI2 (e.g. heparin, citrate, or no anticoagulation).

Types of Outcome Measures

Our primary outcome was circuit lifespan (in hours). Our secondary outcomes were the proportion of bleeding events, thrombotic events, thrombocytopenia, hypotensive events, and mortality rate, as defined by the authors of each study.

Search Methods for Identification of Studies

For this systematic review, we performed a search in Ovid MEDLINE, Ovid Embase, and Cochrane from the inception of the database to June 1, 2022. The search included keywords and controlled vocabulary for prostacyclin, anticoagulation, and extracorporeal support (CRRT, MARS, ECMO) (Supplemental Tables 3-5). We imported the results to Covidence (Covidence Systematic Review Software, Veritas Health Innovation, Melbourne, Australia), which automatically detected duplicates.

Selection of Studies

**Figure 1:**
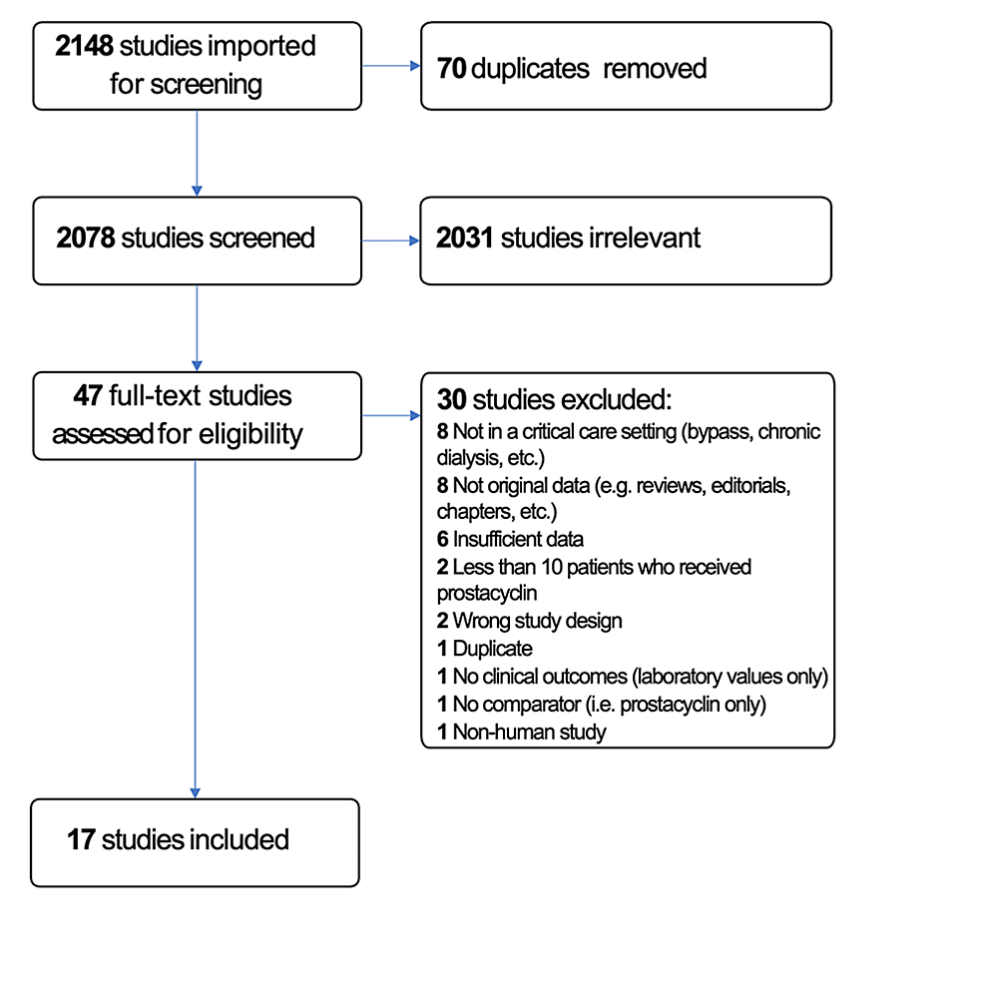
PRISMA diagram detailing the search and selection process applied during the systematic analysis

Five reviewers (NA, AAA, MA, MJ, and OK) independently examined all potential studies and decided on their inclusion in the review (Figure 1). We independently evaluated each study based on its methods and outcomes. We performed this process without blinding of the study authors, institutions, journals of publication, or results. We resolved disagreements through additional evaluation by another author.

Data Extraction and Management

For each study included in the systematic review, two authors independently extracted data. We resolved disagreements through discussion. If required, we contacted study authors to ask for relevant data (e.g. specifying the definition of bleeding or the standard deviation of the average duration of circuit life). The corresponding authors were emailed twice, within a two-week period.

Assessment of Risk of Bias in Included Studies

We evaluated the validity and design characteristics of each study looking for aspects of major potential biases (study participation, prognostic factor measurement, outcome measurement, study confounding, and statistical analysis) [10]. Two authors reviewed and ranked each study’s quality factor separately and defined studies as having a low risk of bias only if they adequately fulfilled all the criteria. 

Assessment of Effect Size

We reported the duration of the extracorporeal filter as mean and standard deviation (SD), and proportions and their 95% confidence interval (95% CI) as the number of patients with the outcomes of interest (bleeding, thrombosis, thrombocytopenia, hypotension, or death) over the total number of patients enrolled in the study. The effect size for continuous variables was the mean difference, whereas the effect size for binary outcomes was the log of the odds ratio. To account for small proportions and interval confidences close to 0, we pooled the individual proportions using arcsin transformation [11]. 

Assuming each study estimated a study-specific true effect, we used random-effect models to pool odds ratios. Such models assume no a priori knowledge about the association between the real, or apparent, intervention effects; the differences between the studies are considered to be random. These models account for heterogeneity, with the center of this distribution describing the average of the effects, and its width describing the degree of heterogeneity. We used the DerSimonian-Laird random-effect method in the presence of significant heterogeneity [12].

We explored the effects of the age categories (adults vs children), the risk of bias (low risk, moderate risk, and high risk), and experimental anticoagulation strategy (prostacyclin alone vs prostacyclin and another anticoagulant), using Harbord’s random-effect meta-regression model [13].

Assessment of Heterogeneity

We explored heterogeneity using the I2 statistic. An I2 statistic higher than 50% represents substantial heterogeneity [14].

Subgroup Analysis

We provided subgroup analyses of outcomes that were significantly different between both arms.

Funnel Plot

We explored funnel plot asymmetry as a surrogate for potential publication biases, solely in randomized controlled trials with significant findings, as the performance of funnel plots in observational studies has not been thoroughly validated [15].

Statistical Analyses

Mean differences (MD), proportion with 95% CI, and random-effects model were undertaken using SPSS (IBM SPSS Statistics for Windows, Version 28.0. Armonk, NY: IBM Corp.). 

Results

Description of Studies

We identified a total of 2,148 references, of which 70 were duplicates and therefore removed from review, leaving a total of 2,078 studies that were screened. Of these, 2,031 studies were irrelevant, leaving 47 full-text articles to be further assessed. Of the 47 article, 25 were excluded because of a non-critical care setting, absence of original data, fewer than 10 patients receiving prostacyclin, duplicates between an abstract and a full text, etc. (Figure 1). In addition, we reached out to the corresponding authors of seven studies, two of which provided the requested data. Five did not respond within the predefined time or declined to provide additional information and were excluded since none of the outcomes of interest were reported in a standardized way (e.g. only the number of circuit changes but not the duration of circuit lifespan was reported). Finally, 17 studies met the eligibility criteria [16-32]. Of these, seven were only available as abstracts [16,21,24-26,29,30].

Overall, the 17 studies reported enrolling 1,333 patients, of which 179 (13.4%) were children [26,30] and 1,154 (86.6%) were adults [16-25,27-29,31,32].

Thirteen studies were observational while four were randomized controlled trials [17,22,23,28]. Sixteen studies enrolled 1261 patients on continuous renal replacement therapy (CRRT) and one study enrolled 72 patients on MARS [25]. No studies enrolled patients on ECMO. 

The studies are described in Table 1.

**Table 1:** Study Description AKI: acute kidney injury; CRRT: continuous renal replacement therapy; ECLS: extracorporeal life support; ICU: intensive care unit; MAP: mean arterial blood pressure; MARS: molecular adsorbent recirculation system; PgE1: prostaglandin E1; PgI2: prostaglandin I2 or prostacyclin; TMP: trans-membrane pressure; RCT: randomized controlled trial; PICU: pediatric intensive care unit.

Studies	Country and Year	Study Design	Abstract vs Full Text	Patient Category	Settings	Sample Size	Type of ECLS	Anticoagulation Strategies	Outcome Definitions				
									Circuit Lifespan	Bleeding	Thrombocytopenia	Hypotension	Mortality
Arcangeli et al., 2010 [22]	Italy, dates not available	Prospective randomized study	Full text	Adults with acute renal failure	Adult	21 patients and 21 filters	CRRT	PgI2 vs heparin	Increase of TMP above 240 mmHg, without parallel venous pressure increase	No data reported	No data reported	Decrease in MAP of 10% from the baseline	No data reported
Balik et al., 2005 [27]	Australia, dates not available	comparative study	Full text	Adults	Adult ICU	33 patients and 95 circuits	CRRT	PgI2+heparin vs citrate	Elevation of TMP above 200 mm Hg without parallel elevation of venous pressure	Not available	Platelet count less than 80,000	Decrease in MAP of 10% of baseline	ICU mortality
Birnbaum et al., 2007 [23]	Germany, dates not available	Prospective randomized pilot study	Full text	Adults with acute renal failure	Adult ICU	20 patients and 20 circuits	CRRT	Heparin vs combined heparin+PgI2	Filter clotting determined by visual evidence of clotting or steady increase of TMP up to 200 cmH2O.	Not available	Platelet count less than 30,000	Not available	ICU mortality
Brookes-Elbaz et al., 2017 [29]	UK, dates not available	Retrospective	Conference paper	Adults with AKI including cardiac (non-ECMO) cases	Adult ICU	240 circuits	CRRT	Citrate vs heparin vs PgI2	Not available	Not available	No data reported	No data reported	No data reported
Davenport et al., 1994 [20]	UK, dates not available	prospective	Full text	Adult patients with combined acute hepatic and renal failure who were at risk of hemorrhage	Adult ICU	17 patients and 65 circuits	CRRT	PgI2 vs heparin	Not available	Hemorrhage that required blood transfusion	Decreased platelet count	Not available	Not available
Fabbri et al., 2010 [28]	Italy, dates not available	Prospective randomized controlled pilot study	Full text	Critically ill adults with acute renal failure	Adult ICU	90 patients	CRRT	Heparin+PgI2 vs heparin	TMP is 250 mmHg and/or blood flow was halted by visible clotting	Severe bleeding: requiring more than 4 units of blood products, or retroperitoneal, intracranial, intraocular, or resulting in death. All other bleeding were considered minimal to moderate.	Not available	Not available	Not available
Goonasekera et al., 2015 [30]	UK, dates not available	Prospective	Conference paper	Pediatric acute liver failure patients	PICU	71 patients	CRRT	PgI2 vs heparin	Not available	Not available	Platelet consumption and need for platelet transfusion	Requirement for fluids/vasopressors	Not available
Herrera-Gutiérrez et al., 2006 [32]	Spain, 1994	Prospective	Full text	Adults with acute renal failure	Adult ICU	389 patients and 1873 circuits	CRRT	PgI2 vs heparin	Not available	No data reported	No data reported	No data reported	No data reported
Hutchison et al., 2016 [24]	UK, dates not available	Prospective	Conference paper	Adults requiring CRRT with no contraindication for regional citrate anticoagulation	Adult ICU	53 patients and 172 circuits	CRRT	Citrate vs PgI2	Not available	No data reported	No data reported	No data reported	No data reported
Journois et al., 1990 [19]	France, dates not available	Retrospective	Full text	Adults with acute renal failure	Adult ICU	9 patients and 36 circuits	CRRT	Heparin vs PgI2+Enoxaparin	Time to reach a third of initial membrane permeability	No data reported	Not available	No data reported	No data reported
Kozek-Langenecker et al., 2002 [17]	Austria, dates not available	double-blinded RCT	Full text	Adults requiring RRT secondary to sepsis or major surgery	Adult ICU	50 patients and 210 circuits	CRRT	Heparin+PgI2 vs heparin+ PgE1	Not available	Significant bleeding is the occurrence of bleeding requiring transfusion of blood products, other types of bleeding considered trivial	No data reported	No data reported	No data reported
Lasorella et al., 2014 [26]	UK, dates not available	Prospective	Conference paper	Children with acute liver failure	PICU	108 patients	CRRT	PgI2 vs heparin	Not available	Not available	platelet consumption	requirement for fluids/vasopressors	Not available
Ostermann et al., 2010 [16]	UK, 2008-2009	Retrospective	Conference paper	Adults requiring CRRT	Adult ICU	288 circuitsepisodes	CRRT	Heparin vs PgI2 vs combined hepain/PgI2	Not available	No data reported	No data reported	No data reported	No data reported
Poply et al., 2014 [21]	UK, dates not available	retrospective	Conference paper	Adults with AKI	Mixed ICU	26 patients and 50 circuits	CRRT	Heparin vs PgI2 vs no anticoagulation	Not available	Not available	Platelet less than 80	No data reported	No data reported
Seller-Perez et al., 2009 [25]	Spain, dates not available	prospective	Conference paper		Adult ICU	43 patients and 43 circuits	MARS	Mixed protocol vs PgI2 vs no anticoagulation	Not available	Not available	No data reported	No data reported	No data reported
Tovey et al., 2013 [18]	UK, 2008-2009	Retrospective	Full text	Adults with severe AKI requiring CRRT	Adult ICU	309 patients and 2059 circuits	CRRT	Heparin vs PgI2 vs citrate	Premature filter clotting excluding elective discontinuation	No data reported	No data reported	No data reported	No data reported
Windelov et al., 2010 [31]	Denmark, 2009-2008	retrospective	Full text	Adults with AKI requiring CRRT	Adult ICU	94 patients	CRRT	Heparin vs PgI2	No data reported	No data reported	Not available	No data reported	Mortality at 30, 90, and 365 days

Anticoagulation Strategies

Thirteen of the 17 studies (77%) compared prostacyclin to heparin [16,18-23,26,28-32], and four (24%) compared prostacyclin to citrate [18,24,27,29]. Of note, five (29%) compared a combination of prostacyclin and heparin to heparin alone [16,19,23,27,28], and five studies (29%) had more than two groups [16,18,21,25,29].

Assessment of the Risk of Bias

The risk of bias was low in nine studies [18,20-23,25,27,28,31], moderate in seven studies [16,17,19,24,26,30,32], and high in one study [29]. The full assessment is shown in Table 2. 

**Table 2:** Assessment of Studies ⊕⊕⊕ represents high quality (i.e. low risk of bias), ⊕⊕⊖ moderate quality (i.e. moderate risk of bias), ⊕⊖⊖ low quality (i.e. high risk of bias), and ⊖⊖⊖ very low quality (i.e. very high risk of bias)

Studies	Study Participation	Study Attrition	Prognostic Factor Measurement	Outcome Measurement	Study Confounding	Statistical Analysis and Presentation	Overall Quality
Arcangeli et al., 2010 [22]	⊕⊕⊕	⊕⊕⊕	⊕⊕⊕	⊕⊕⊕	⊕⊕⊕	⊕⊕⊕	⊕⊕⊕
Balik et al., 2005 [27]	⊕⊕⊕	⊕⊕⊕	⊕⊕⊕	⊕⊕⊕	⊕⊕⊕	⊕⊕⊕	⊕⊕⊕
Birnbaum et al., 2007 [23]	⊕⊕⊖	⊕⊕⊕	⊕⊕⊕	⊕⊕⊕	⊕⊕⊕	⊕⊕⊕	⊕⊕⊕
Brookes-Elbaz et al., 2017 [29]	⊕⊖⊖	⊕⊕⊕	⊕⊕⊕	⊕⊕⊕	⊕⊖⊖	⊕⊖⊖	⊕⊖⊖
Davenport et al., 1994 [20]	⊕⊕⊖	⊕⊕⊕	⊕⊕⊖	⊕⊕⊕	⊕⊕⊕	⊕⊕⊕	⊕⊕⊕
Fabbri et al., 2010 [28]	⊕⊕⊕	⊕⊕⊕	⊕⊕⊖	⊕⊕⊕	⊕⊕⊕	⊕⊕⊕	⊕⊕⊕
Goonasekera et al., 2015 [30]	⊕⊕⊖	⊕⊕⊕	⊕⊕⊕	⊕⊕⊕	⊕⊕⊖	⊕⊕⊕	⊕⊕⊖
Herrera-Gutiérrez et al., 2006 [32]	⊕⊕⊕	⊕⊕⊕	⊕⊕⊖	⊕⊕⊕	⊕⊕⊖	⊕⊖⊖	⊕⊕⊖
Hutchison et al., 2016 [24]	⊕⊕⊕	⊕⊕⊕	⊕⊕⊕	⊕⊕⊕	⊕⊖⊖	⊕⊖⊖	⊕⊕⊖
Journois et al., 1990 [19]	⊕⊕⊕	⊕⊕⊕	⊕⊕⊕	⊕⊕⊕	⊕⊕⊖	⊕⊖⊖	⊕⊕⊖
Kozek-Langenecker et al., 2002 [17]	⊕⊕⊕	⊕⊕⊕	⊕⊕⊕	⊕⊕⊕	⊕⊖⊖	⊕⊕⊕	⊕⊕⊖
Lasorella et al., 2014 [26]	⊕⊕⊖	⊕⊕⊕	⊕⊕⊕	⊕⊕⊖	⊕⊖⊖	⊕⊕⊕	⊕⊕⊖
Ostermann et al., 2010 [16]	⊕⊕⊕	⊕⊕⊕	⊕⊖⊖	⊕⊕⊕	⊕⊕⊖	⊕⊖⊖	⊕⊕⊖
Poply et al., 2014 [21]	⊕⊕⊕	⊕⊕⊕	⊕⊕⊕	⊕⊕⊕	⊕⊕⊖	⊕⊕⊕	⊕⊕⊕
Seller-Perez et al., 2009 [25]	⊕⊕⊕	⊕⊕⊕	⊕⊕⊕	⊕⊕⊕	⊕⊕⊕	⊕⊕⊕	⊕⊕⊕
Tovey et al., 2013 [18]	⊕⊕⊕	⊕⊕⊕	⊕⊕⊕	⊕⊕⊕	⊕⊕⊖	⊕⊕⊕	⊕⊕⊕
Windelov et al., 2010 [31]	⊕⊕⊕	⊕⊕⊕	⊕⊕⊕	⊕⊕⊕	⊕⊕⊕	⊕⊕⊕	⊕⊕⊕

Circuit lifespan

**Figure 2:**
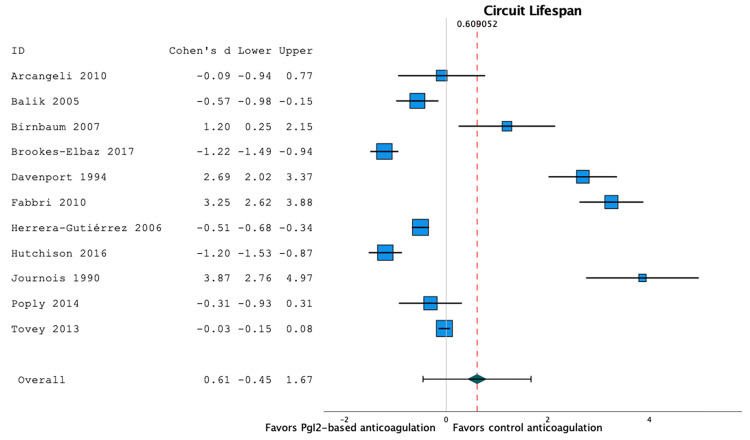
Forest plot of the filter lifespan according to the anticoagulation strategy There was no significant difference in the circuit lifespan between prostacyclin-based anticoagulation strategies and heparin- or citrate-based anticoagulation strategies (mean difference 2.5 hour (95%CI -12.0;16.9), p=0.74, I2=0.99, n=4,003 circuits) [16,18–24,27–29,32].

Twelve studies reported circuit lifespan [16,18-24,27-29,32]. However, one study [16] reported circuit lifespan without providing a standard deviation and was therefore included in systematic review (see Table 1) but not in the meta-analysis. Among the remaining 11 studies, the mean circuit lifespan was 29.7 hours in the patients in prostacyclin-based anticoagulation series (95%CI 21.8;37.6, I2=98.7, n=974 circuits), and 27.2 hours in the patients in heparin- or citrate-based anticoagulation series (95%CI 19.4;34.1, I2=99.1, n=3029 circuits). There was no significant difference in the circuit lifespan between prostacyclin-based anticoagulation strategies and heparin- or citrate-based anticoagulation strategies: mean difference 2.5 hour (95%CI -12.0;16.9), p=0.74, I2=0.99, n=4,003 circuits (Figure 2).

A random-effect meta-regression model to evaluate the effect of co-variables showed that circuit lifespan was not associated with quality of evidence (p=0.83) and prostacyclin strategy (p=0.81). As all these studies enrolled adults, the effect of the age could not be evaluated in this regression model.

Bleeding Events

**Figure 3:**
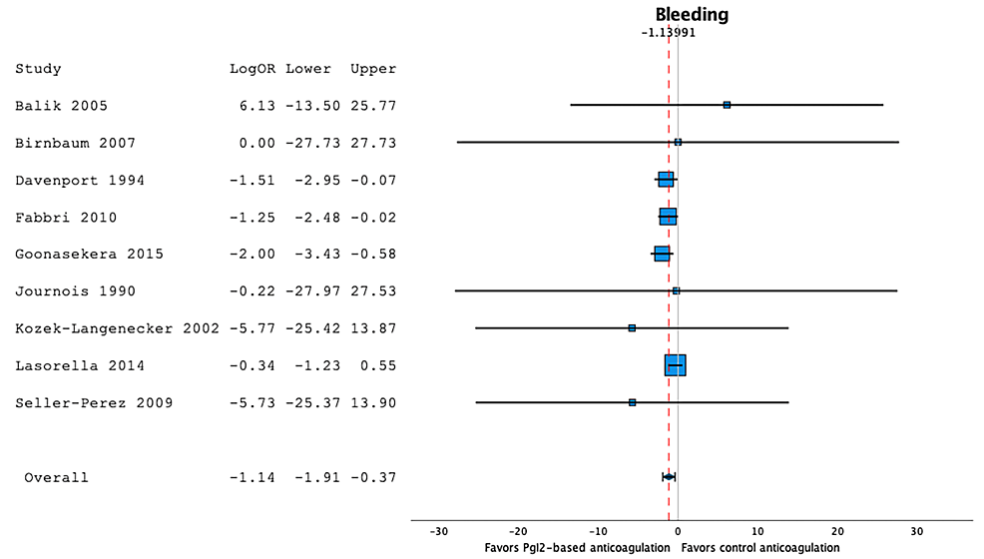
Forest plot of the bleeding events according to the anticoagulation strategy Prostacyclin-based anticoagulation strategies were associated with a significant decrease in the proportion of bleeding when compared to heparin- or citrate-based anticoagulation strategies (LogOR -1.14 (95%CI -1.91;-0.37), p<0.001, I2=0.19, n=470) [17,19,20,23,25–28,30].

Nine studies reported bleeding events [17,19,20,23,25-28,30]. Bleeding occurred in 9.5% of the patients in prostacyclin-based anticoagulation series (95%CI 4.3;16.1, I2=45.8, n=257), and in 17.1% of the patients in heparin- or citrate-based anticoagulation series (95%CI 8.9;26.9, I2=52.1, n=213). A random-effect model showed that prostacyclin-based anticoagulation strategies were found to be associated with a significant decrease in the proportion of bleeding when compared to heparin- or citrate-based anticoagulation strategies: LogOR -1.14 (95%CI -1.91;-0.37), p<0.001, I2=0.19, n=470 (Figure 3).

A random-effect meta-regression model evaluating the effect of co-variables showed that the proportion of bleeding events was not independently associated with quality of evidence (p=0.49), age category (p=0.55), or prostacyclin strategy (p=0.95).

**Figure 4:**
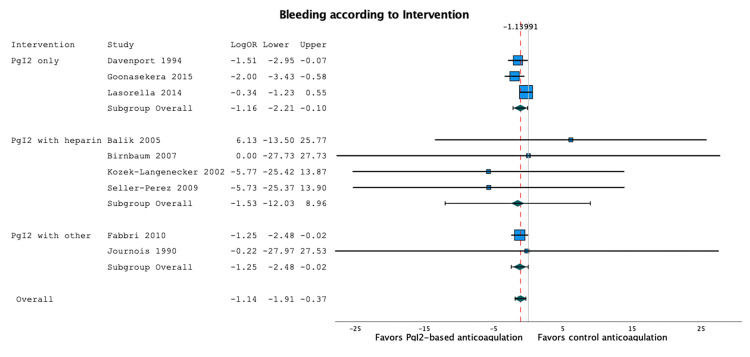
Subgroup forest plot of the bleeding events, according to the intervention (PgI2 alone or in combination with another anticoagulant). The results are similar across the subgroups [17,19,20,23,25–28,30].

Figure 4 is the subgroup forest plot comparing the two prostacyclin strategies (i.e. prostacyclin alone vs with heparin). 

**Figure 5:**
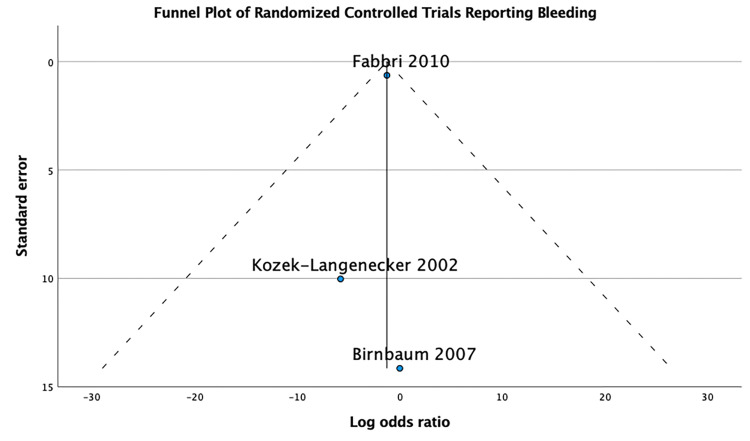
Funnel plot of the bleeding events. [17,23,28].

The funnel plot performed for the three randomized control trials that reported bleeding outcome [17,23,28] is symmetrical, which doesn’t suggest publication bias (Figure 5).

Thrombotic Events

**Figure 6:**
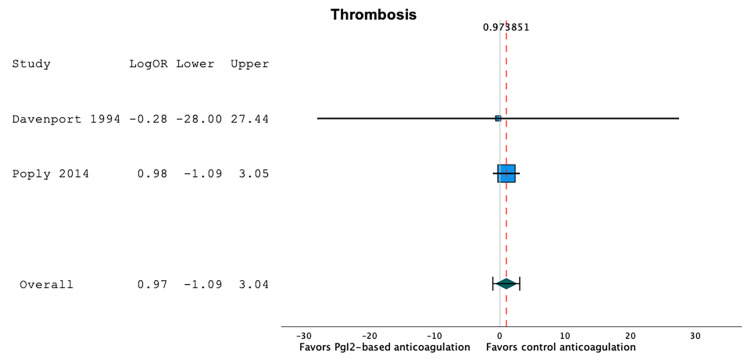
Forest plot of the thrombotic events according to the anticoagulation strategy. Prostacyclin-based anticoagulation strategies were not associated with a significant difference in the proportion of thrombotic events when compared to heparin- or citrate-based anticoagulation strategies (LogOR 0.97 (95%CI -1.09;3.04), p=0.35, I2=0.0, n=115) [20,21].

Two studies reported thrombotic events [20,21]. Thrombotic events occurred in 3.6% of the patients in prostacyclin-based anticoagulation series (95%CI 0.0;37.1, I2=77.9, n=51), and in 2.2% of the patients in heparin- or citrate-based anticoagulation series (95%CI 0.0;10.2, I2=37.9, n=64). A random-effect model showed that prostacyclin-based anticoagulation strategies were not associated with a significant difference in the proportion of thrombotic events when compared to heparin- or citrate-based anticoagulation strategies: LogOR 0.97 (95%CI -1.09;3.04), p=0.35, I2=0.0, n=115 (Figure 6). There were too few studies to be able to use a random-effect meta-regression model.

Hypotensive Events

**Figure 7:**
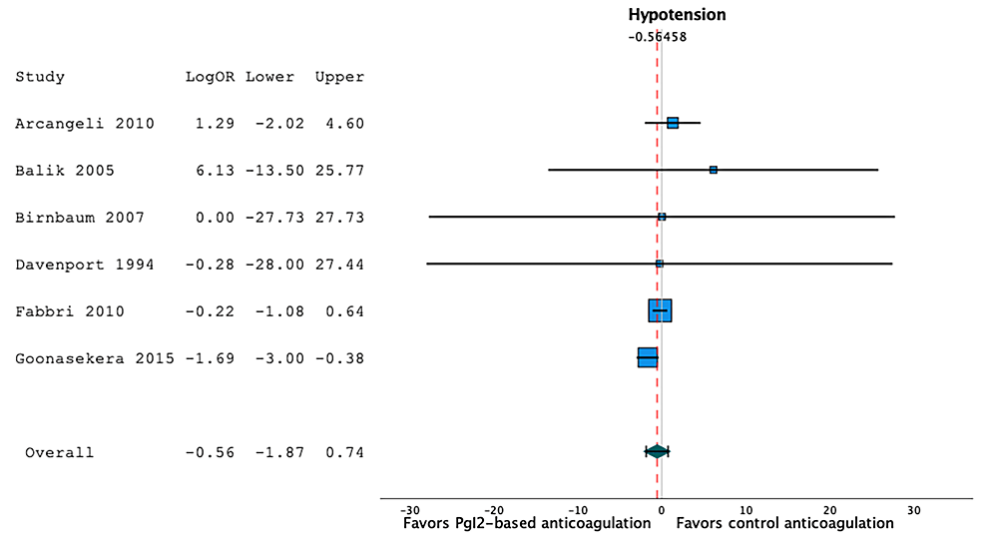
Forest plot of the hypotensive events according to the anticoagulation strategy. Prostacyclin-based anticoagulation strategies were not associated with a significant difference in the proportion of hypotensive events when compared to heparin- or citrate-based anticoagulation strategies (LogOR -0.56 (95%CI -1.87;0.74), p=0.40, I2=0.35, n=299) [20,22,23,27,28,30].

Six studies reported hypotensive events [20,22,23,27,28,30]. Hypotensive events occurred in 13.4% of the patients in prostacyclin-based anticoagulation series (95%CI 0.6;35.3, I2=89.3, n=165), and in 11.0% of the patients in heparin- or citrate-based anticoagulation series (95%CI 0.0;36.9, I2=91, n=134). A random-effect model showed that prostacyclin-based anticoagulation strategies were not associated with a significant difference in the proportion of hypotensive events when compared to heparin- or citrate-based anticoagulation strategies: LogOR -0.56 (95%CI -1.87;0.74), p=0.40, I2=0.35, n=299 (Figure 7).

 

Using a random-effect meta-regression model to evaluate the effect of co-variables, we observed that the proportion of hypotensive events was not independently associated with age categories (p=0.41) or prostacyclin strategy (p=0.91). As all but one study had a low risk of bias, the effect of the quality of evidence could not be evaluated in this regression model.

Thrombocytopenic Events

Only one study reported thrombocytopenic events [27]. Thrombocytopenic events occurred in 23.5% of the patients in the prostacyclin-based anticoagulation series (95%CI 7.8;50.2, n=17), and in 0% of the patients in heparin- or citrate-based anticoagulation series (95%CI 0.0;25.4, n=15).

Mortality Rate

**Figure 8:**
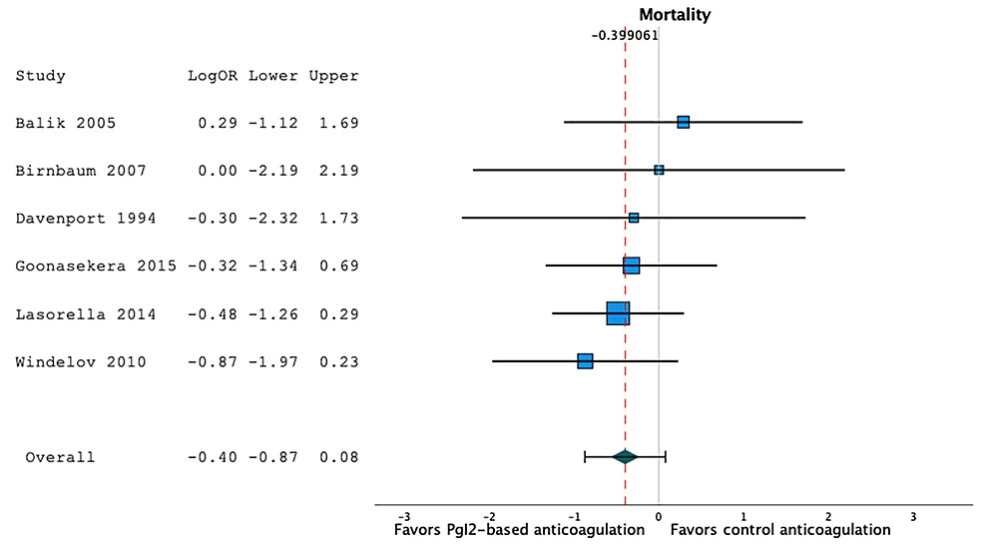
Forest plot of the mortality rates according to the anticoagulation strategy. Prostacyclin-based anticoagulation strategies were not associated with a significant difference in the mortality rate when compared to heparin- or citrate-based anticoagulation strategies (LogOR -0.40 (95%CI -0.87;0.08), p=0.10, I2=0.00, n=390) [20,23,26,27,30,31].

Six studies reported mortality [20,23,26,27,30,31]. The mortality rate was 26.3% in prostacyclin-based anticoagulation series (95%CI 13.6;41.2, I2=76.4, n=198), and 32.7% in heparin- or citrate-based anticoagulation series (95%CI 18.6;48.4, I2=76.3, n=192). Using a random-effect model, we observed that prostacyclin-based anticoagulation strategies were not associated with a significant difference in the mortality rate when compared to heparin- or citrate-based anticoagulation strategies: LogOR -0.40 (95%CI -0.87;0.08), p=0.10, I2=0.00, n=390 (Figure 8).

Using a random-effect meta-regression model to evaluate the effect of co-variables, we observed that the mortality rate was not independently associated with age categories (p=0.62) or prostacyclin strategy (p=0.45). As all but one study had a low risk of bias, the effect of the quality of evidence could not be evaluated in this regression model.

Discussion

This meta-analysis of 17 studies, that enrolled 1,333 patients requiring extracorporeal support (mostly CRRT), showed a significant reduction in bleeding events in the prostacyclin-based strategy when compared to other strategies. There were no statistically significant differences in circuit lifespan, hypotensive events, thrombotic events, thrombocytopenia, and mortality. 

Circuit failure, which is defined as a premature failure of the circuit before the expected lifespan, might occur because of the complex blood-circuit biocompatibility, patient’s critical condition, inflammation, size and position of vascular access, and efficiency of anticoagulation [33]. Circuit failure might lead to suboptimal treatment efficacy [34], and increased transfusion requirements [35]. Although the available literature about the economic impact of premature circuit failure is scarce, a shorter circuit lifespan is likely associated with higher costs. While our results failed to show an improved circuit lifespan, they do suggest a decreased risk of bleeding. Bleeding complications can be secondary to the patient’s underlying coagulopathy, complications of vascular access, or secondary to the need for anticoagulation. While heparin-induced bleeding has been reported to be as high as 30% on CRRT [36], our meta-analysis showed the risk of bleeding to be 17% in the control groups (heparin or citrate), and only 9.5% in patients who received prostacyclin. Therefore, prostacyclin-based anticoagulation strategies could decrease bleeding events without increasing thrombotic events or decreasing the circuit lifespan. 

Limitations

While this meta-analysis is the first to evaluate prostacyclin in all types of extracorporeal support and in both critically ill adults and children, certain limitations must be acknowledged. First, most of the included studies were observational in nature, which might have led to a selection bias. Second, as for all systematic reviews, there might have been a publication bias. Third, only two studies enrolled pediatric patients [26,30]. Fourth, there was heterogeneity among the intervention, since some studies used a prostacyclin dose of 1 ng/kg/min [23] to 10 ng/kg/min [27]. In addition, the co-anticoagulation was also heterogeneous, with some studies using heparin at various doses, and others using citrate. Fifth, the outcome definitions were also heterogeneous, as some studies defined circuit failure based on different cut-off trans-membrane pressures, while others used visible circuit clots. Finally, most of the studies enrolled patients on CRRT in which blood flow rate and circuit characteristics factor in CRRT failure and circuit clotting (apart from MARS that uses a primary CRRT device).This prevents us from extrapolating these results to other modalities, such as ECMO or plasma exchange therapy. 

## Conclusions

In conclusion, in a systematic review and meta-analysis of 17 studies, prostacyclin-based anticoagulation was associated with fewer bleeding events, but with similar circuit lifespans, thrombotic events, hypotensive events, and mortality rates. Our study has some limitations, such as heterogeneity in the interventions (prostacyclin alone vs prostacyclin with other anticoagulants) and in the outcome definitions. In addition, nearly all studies enrolled patients on continuous renal replacement therapy, which prevents our results to be extrapolated to other modalities of extracorporeal support, such as extracorporeal membrane oxygenation. The potential benefits of prostacyclin-based anticoagulation should be further explored in large randomized controlled trials to investigate the optimal dose as well as the applicability to other extracorporeal support modalities.

## Appendices

Search Strategy

**Table 3:** Ovid Medline Search Strategy

1	(Prostacyclin or epoprostenol or prostaglandin I2 or PGI2 or iloprost or cycloprostin or cyclo-prostin or prostaglandin i 2 or caripul or flolan or act 385781a).mp. or exp Epoprostenol/
2	Renal Replacement Therapy/ or exp Continuous Renal Replacement Therapy/ or exp Hemofiltration/ or exp Hemoperfusion/ or exp Hybrid Renal Replacement Therapy/ or exp Intermittent Renal Replacement Therapy/ or exp Renal Dialysis/ or (renal replacement therapy or hemofiltration or hemodialysis or kidney replacement therapy or dialysis).mp.
3	exp Extracorporeal Membrane Oxygenation/ or (exp Oxygenators, Membrane/ and (extracorporeal or "extra corporeal").mp.) or exp Extracorporeal Circulation/ or exp Heart-Lung Machine/ or (extracorporeal membrane oxygenation or extracorporeal life support or veno-arterial extra-corporeal membrane oxygenation or veno-venous extra-corporeal membrane oxygenation or ECMO or ECLS).mp.
4	((extracorporeal or "extra corporeal" or "extra-corporeal") and membra* and oxygen*).mp.
5	((extracorporeal adj3 oxygenation) or ("extra corporeal" adj3 oxygenation) or (extrapulmonary adj3 oxygenation) or ("extra pulmonary" adj3 oxygenation)).mp.
6	3 or 4 or 5
7	2 or 6
8	1 and 7

**Table 4:** Ovid Embase Search Strategy

1	(Prostacyclin or epoprostenol or prostaglandin I2 or PGI2 or iloprost or cycloprostin or cyclo-prostin or prostaglandin i 2 or caripul or flolan or act 385781a).mp. or exp prostacyclin/
2	exp extracorporeal oxygenation/ or (extracorporeal membrane oxygenation or extracorporeal life support or veno-arterial extra-corporeal membrane oxygenation or veno-venous extra-corporeal membrane oxygenation or ECMO or ECLS).mp. or ((extracorporeal or "extra corporeal" or "extra-corporeal") and membra* and oxygen*).mp.
3	exp extracorporeal circulation/ or exp heart lung machine/ or (exp membrane oxygenator/ and (extracorporeal or "extra corporeal").mp.)
4	((extracorporeal adj3 oxygenation) or ("extra corporeal" adj3 oxygenation) or (extrapulmonary adj3 oxygenation) or ("extra pulmonary" adj3 oxygenation)).mp.
5	2 or 3 or 4
6	renal replacement therapy/ or exp continuous renal replacement therapy/ or extended daily dialysis/ or exp hemodiafiltration/ or exp hemodialysis/ or exp hemofiltration/ or intermittent renal replacement therapy/
7	(renal replacement therapy or hemofiltration or hemodialysis or kidney replacement therapy or dialysis).ti,ab,kw.
8	6 or 7
9	5 or 8
10	1 and 9

**Table 5:** Cochrane Search Strategy

#1	MeSH descriptor: [Epoprostenol] explode all trees
#2	Prostacyclin OR epoprostenol OR "prostaglandin I2" OR PGI2 OR iloprost OR cycloprostin OR cyclo-prostin OR "prostaglandin i 2" OR caripul OR flolan OR "act 385781a"
#3	#1 OR #2
#4	extracorporeal membrane oxygenation or extracorporeal life support or veno-arterial extra-corporeal membrane oxygenation or veno-venous extra-corporeal membrane oxygenation or ECMO or ECLS or ((extracorporeal or "extra corporeal" or "extra-corporeal") and membra* and oxygen*)
#5	MeSH descriptor: [Heart-Lung Machine] explode all trees
#6	MeSH descriptor: [Extracorporeal Membrane Oxygenation] explode all trees
#7	MeSH descriptor: [Extracorporeal Circulation] explode all trees
#8	MeSH descriptor: [Oxygenators, Membrane] explode all trees
#9	#8 AND (extracorporeal or "extra corporeal")
#10	#4 or #5 or #6 or #7 or #9
#11	renal replacement therapy or hemofiltration or hemodialysis or "kidney replacement therapy" or dialysis
#12	MeSH descriptor: [Renal Replacement Therapy] explode all trees
#13	#11 or #12
#14	#10 or #13
#15	#3 AND #14
